# Oncogenes and tumor suppressor genes: comparative genomics and network perspectives

**DOI:** 10.1186/1471-2164-16-S7-S8

**Published:** 2015-06-11

**Authors:** Kevin Zhu, Qi Liu, Yubo Zhou, Cui Tao, Zhongming Zhao, Jingchun Sun, Hua Xu

**Affiliations:** 1 Graduate School of Biomedical Sciences, The University of Texas Health Science Center at Houston, Houston, TX 77030, USA; 2 Department of Biomedical Informatics, Vanderbilt University, Nashville, TN 37203, USA; 3 National Center for Drug Screening, Shanghai Institute of Materia Medica, Chinese Academy of Sciences, Shanghai, People's Republic of China; 4 School of Biomedical Informatics, The University of Texas Health Science Center at Houston, Houston, TX 77030, USA

## Abstract

**Background:**

Defective tumor suppressor genes (TSGs) and hyperactive oncogenes (OCGs) heavily contribute to cell proliferation and apoptosis during cancer development through genetic variations such as somatic mutations and deletions. Moreover, they usually do not perform their cellular functions individually but rather execute jointly. Therefore, a comprehensive comparison of their mutation patterns and network properties may provide a deeper understanding of their roles in the cancer development and provide some clues for identification of novel targets.

**Results:**

In this study, we performed a comprehensive survey of TSGs and OCGs from the perspectives of somatic mutations and network properties. For comparative purposes, we choose five gene sets: TSGs, OCGs, cancer drug target genes, essential genes, and other genes. Based on the data from Pan-Cancer project, we found that TSGs had the highest mutation frequency in most tumor types and the OCGs second. The essential genes had the lowest mutation frequency in all tumor types. For the network properties in the human protein-protein interaction (PPI) network, we found that, relative to target proteins, essential proteins, and other proteins, the TSG proteins and OCG proteins both tended to have higher degrees, higher betweenness, lower clustering coefficients, and shorter shortest-path distances. Moreover, the TSG proteins and OCG proteins tended to have direct interactions with cancer drug target proteins. To further explore their relationship, we generated a TSG-OCG network and found that TSGs and OCGs connected strongly with each other. The integration of the mutation frequency with the TSG-OCG network offered a network view of TSGs, OCGs, and their interactions, which may provide new insights into how the TSGs and OCGs jointly contribute to the cancer development.

**Conclusions:**

Our study first discovered that the OCGs and TSGs had different mutation patterns, but had similar and stronger protein-protein characteristics relative to the essential proteins or control proteins in the whole human interactome. We also found that the TSGs and OCGs had the most direct interactions with cancer drug targets. The results will be helpful for cancer drug target identification, and ultimately, understanding the etiology of cancer and treatment at the network level.

## Background

Cancer is the second disease leading to death worldwide [1]. It consists of more than 100 different diseases with diverse risk factors. Among these risk factors, genetic alternations play critical roles in the pathogenesis of the disease and provide fundamental clues for the identification of drug targets and the development of novel drugs [2-6]. Recently, several large-scale cancer genome projects produced multi-dimensional genome-wide big data such as The Cancer Genome Atlas (TCGA) [7], the Wellcome Trust Sanger Institute's Cancer Genome Project [8], and the International Cancer Genome Consortium (ICGC) [9]. These genome-wide data have dramatically advanced cancer research, especially in terms of its genetics and genomics [10], which enhances the accuracy and coverage of the identification of cancer-related genes that could drive or protect cancer development. However, though these large-scale sequencing data discovered thousands of mutations, they have not so far identified novel drug targets besides these previously identified [11]. Therefore, developing novel approaches to revealing the signal buried under the big data to discover the novel drug targets is necessary and critical for the development of effective treatment for cancer.

Among these cancer-related genes identified by high-throughput sequencing and small-scale traditional approaches, two classes of genes - tumor suppressor genes (TSGs) and oncogenes (OCGs) - have been attracted much attention. Numerous studies demonstrated these genetic alternations involve the gain-of-function of OCGs together with the loss-of-function of TSGs determine the cell cycle processes that control the tumor formation and development [12,13]. Recently, protein-protein interaction (PPI) network based on computational methods have been used to identify disease-specific genes, modules, and cancer-subtype subnetworks [14-16]. Therefore, we hypothesized that comparative investigations of TSG and OCG mutation patterns and network properties would provide a number of novel insights into their functions in the tumorigenesis, which further offers valuable information for identification of novel drug targets for drug development.

As numerous genetic and genomic data in cancer become available, the list of OCGs and TSGs has been expanded through molecular, cellular, genomic, and computational studies including non-coding RNA genes [17,18]. Considering the gain-of-function of OCG mutations and loss-of-function of TSG mutations, TSGs and OCGs may be involved in the regulation of cellular functions in a yin-yang fashion [19]. For example, our previous study has shown that they have distinct and competitive regulatory patterns in ovarian cancer [20]. Furthermore, OCG mutations are usually dominant so that one mutant copy is enough to start switching on a cellular activity. TSG mutations tend to be recessive, so that they should follow the famous Knudson's 'two-hit hypothesis': that both copies of tumor suppressor genes need mutate to cause loss of function. However, more and more evidence shows that even partial inactivation of TSGs could critically contribute to tumorigenesis [21]. Additionally, some genes' function could be switched between OCGs and TSGs, depending on the situation. Current therapeutic applications have shown that targeting OCGs and their related pathways is promising for developing novel drugs, including antibodies and small synthetic molecules [22]. Therefore, further understanding of OCGs and TSGs in the terms of networks will provide novel insights into the their functions in the tumorigenesis. However, to our knowledge, there is no report that systematically investigates their relationships.

Thus, in this study, we compared five sets of proteins encoded by five sets of genes (TSGs, OCGs, drug target genes, essential genes, and other genes) with the perspectives of genomics and protein networks. We compared them using the somatic mutations from TCGA Pan-Cancer project [23] and network properties in human PPI networks [24]. Based on the genetic data from Pan-Cancer project, we found that TSGs had the highest mutation frequency in most tumor types and the OCGs second. For the network properties, relative to target proteins, essential proteins, and other proteins, both TSG and OCG proteins tended to have higher degrees, higher betweenness, lower clustering coefficients, and shorter shortest-path distances. In addition, both TSG and OCG proteins tended to have direct interactions with cancer drug target proteins. We further generated a TSG-OCG network and found that TSGs and OCGs connected strongly with each other. Our study first revealed that the OCGs and TSGs had different mutation patterns, but had similar and stronger protein-protein characteristics relative to the essential proteins or control proteins in the whole human interactome.

## Materials and methods

### Somatic mutations of the Cancer Genome

To explore the somatic mutation patterns, we obtained the somatic mutations from Supplementary Table 2 published by one Pan-Cancer analysis of TCGA project [23]. The study presents the data and analytical results for point mutations and small insertions/deletions from 3,281 tumours across 12 tumour types. The 12 tumours included bladder urothelial carcinoma (BLCA), breast adenocarcinoma (BRCA), colon and rectal adenocarcinoma (COAD/READ), glioblastoma (GBM), head and neck squamous cell carcinoma (HNSC), kidney renal clear cell carcinoma (KIRC), acute myeloid leukemia (LAML), lung adenocarcinoma (LUAD), lung squamous cell carcinoma (LUSC), ovarian cancer (OV), and uterine corpus endometrioid carcinoma (UCEC).

### Human PPIs

To study the network properties of gene sets, we utilized the most recent version of the human PPI data from the Protein Interaction Network Analysis platform (PINA v2.0) [24]. After mapping the human protein IDs to their official gene symbols, we culled out the redundant connections and the self-interactions. The interaction network contains 12,978 nodes corresponding to human 12,978 genes and 101,219 edges.

### Gene sets

In this study, we choose TSGs and OCGs with high confidence from Davioli et al. [17]. Each set of TSGs and OCGs contains 50 genes that were selected from the Cancer Gene Census and have been implicated in tumorigenesis by experimental evidence in the literature [25].

To get cancer-related drugs, we utilized the Anatomical Therapeutic Chemical (ATC) Classification codes L01 (Antineoplastic Agents) to obtain the cancer drugs from DrugBank [26]. We first downloaded the data from the DrugBank database (version 4.0, June 2014) and extracted the drug-related information, such as the "Name," "Drug Targets," and "ATC Codes." Consequently, we obtained a total of 115 cancer drugs with their drug targets. These drug targets could map to 171 gene official symbols. We regarded them as cancer drug target genes.

For comparative purposes, besides TSGs, OCGs, and drug target genes, we included essential genes and other genes as controls. For the essential genes, we utilized the gene list that were predicted at the cellular level [17]. The other genes contained genes encoding proteins in the PINA PPI data set after excluding the OCGs, TSGs, targets, and essential genes. Overall, we investigated five gene sets in this study: TSGs, OCGs, target genes, essential genes, and others.

### Network properties

To explore network properties of these five sets of genes, we calculated four basic and important network properties: degree, betweenness, clustering coefficient, and shortest-path distance [27,28]. The degree (connectivity) of a node *A *is the number of other nodes that are directly connected to *A *by an edge. These nodes are neighbors of node A. A node with a higher degree will have a higher number of neighbors. The betweenness of a node *A *describes how many shortest paths between any two pairs in the network will pass through *A*. The clustering coefficient represents the ratio of the number of connections that occur between the immediate neighbors of *A *compared to the maximum number of connections that could occur among them. The shortest-path distance between two pair of nodes *A *and *B *is the smallest number nodes that must be passed through to get from *A *to *B*. This means that if *A *and *B *are neighbors, the shortest-path distance between them would be one. Given sets of nodes, we calculated the shortest-paths from a set of interest nodes to all other nodes in the network. Moreover, we calculated the shortest-path distances between target proteins to other interest gene set to measure their interrelationship. At the each distance, we calculated the proportion of interest proteins.

### Subnetwork generation

To better understand the interactions between OCGs and TSGs, we generated a subnetwork that contains OCGs and TSGs using the GenRev program [29] (version 1.0.1). Given a network and a set of interest nodes, GenRev enables calculate a subnetwork containing the interest nodes and non-interest nodes. The interest nodes are terminal nodes while the non-interest nodes are linker nodes that become part of the subnetwork based on the algorithm's criteria. GenRev offers three algorithms for generating subnetworks: the Klein-Ravi algorithm, the limited k-walk algorithm, and a heuristic local search algorithm. In this study, we utilized the Klein-Ravi algorithm to generate a node-weighted Steiner tree subnetwork. The algorithm enables to intertwine as many terminal nodes as possible through non-interest nodes (linkers) by calculating the shortest-path distance [30].

## Results

### TSGs have the highest frequency of mutations

In this study, we choose the 50 TSGs, 50 OCGs, and 145 essential genes, 171 target genes, and 12,315 other genes for investigation of mutation patterns. To compare the mutation frequencies of the tumor samples among the five gene sets, we performed the Kolmogorov-Smirnov (K-S) tests [31].

**Figure 1:**
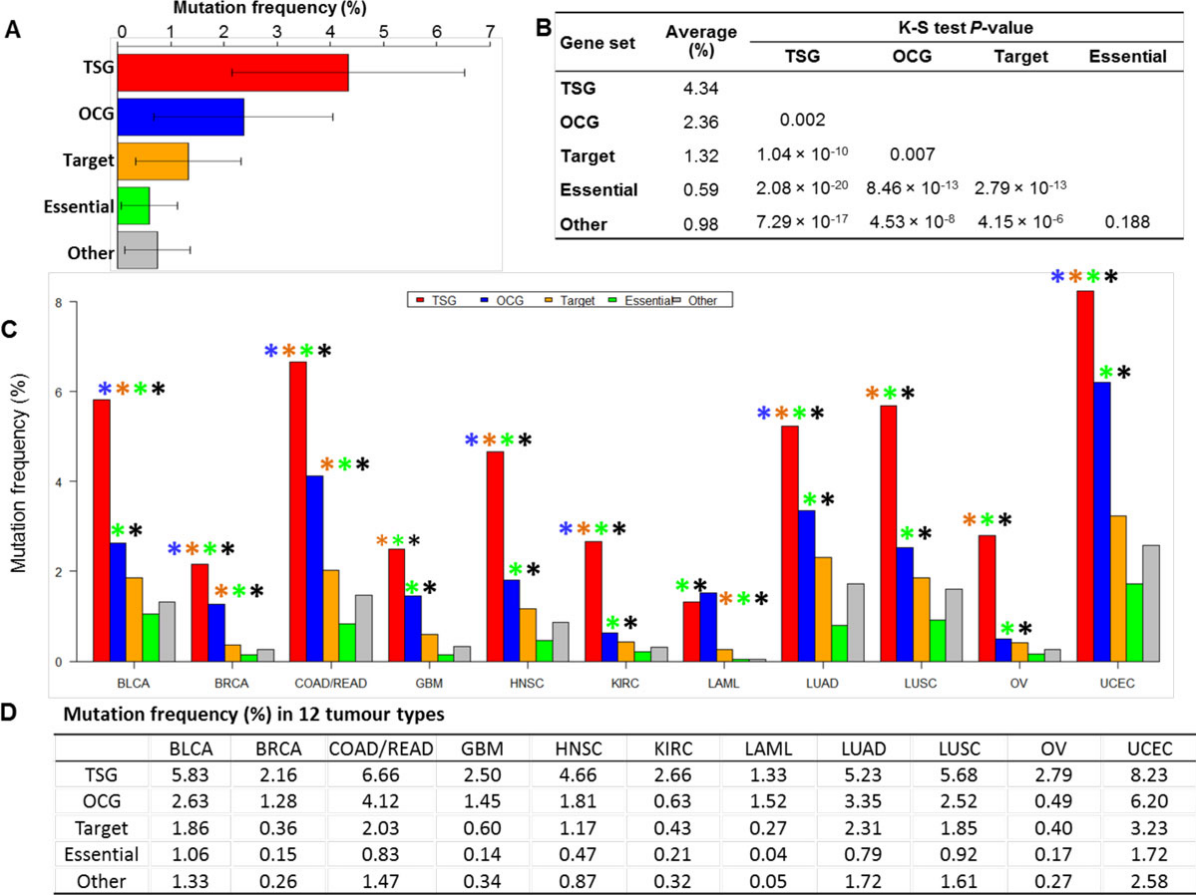
**Percentage comparison of Pan-Cancer samples mutated in five gene sets (A and B) and across 12 tumor types (C and D)**. In Figure D, one star indicates a *P-value *less than 0.05 based on the Kolmogorov-Smirnov (K-S) test between the two gene sets. The star color indicates the corresponding gene sets. For example, in BLCA, the top of the TSG bar has four stars, which indicates that the percentage of samples with mutations in the TSG gene set was significantly higher than that of OCG genes (blue star), target genes (red star), essential genes (green star), and other genes (gray star). 'TSG' represents the tumor suppressor genes, 'OCG' represents the oncogenes, 'Target' represents the genes encoding cancer drug targets, 'Essential' represents the essential genes, and 'Other' represents the other genes with mutation data that are part of the PPI data.

Figure 1A shows a comparison of a general mutation percentage of all samples in each gene set, and Figure 1B contains the average values and *P*-values of five gene sets. The TSGs had the highest average mutation frequency (4.34%), which was significantly higher than that of OCGs (2.36%, *P *= 0.002), target genes (1.32%, *P *= 1.04 × 10^-10^), essential genes (0.59%, *P *= 2.08 ×10^-20^), and other genes (0.98%, *P *= 7.29 × 10^-17^). The OCGs had the second highest average mutation frequency (2.36%), which was significantly higher than that of target genes (*P *= 0.007), essential genes (*P *= 8.46 ×10^-13^), and other genes (*P *= 4.53 × 10^-17^), respectively. The target genes had the third highest average mutation frequency (1.32%), which was significantly higher than that of essential genes (*P *= 2.79 × 10^-13^) and other genes (*P *= 4.15 × 10^-6^). Interestingly, the essential gene had the lowest mutation frequency among the five gene sets.

We further examined the mutation frequency in the five gene sets across the 12 cancer types (Figure 1C and Figure 1D). The mutation frequency in the TSGs was significantly higher than that of all other tumor types except for GBM, LAML, LUSC, and OV (*p* <0.05). LAML had the lowest average mutation frequency (1.33%) and UCEC the highest (8.23%). The mutation frequency in the OCGs was significantly higher than that of essential genes and that of other genes (*p *<0.05), respectively. Only in BRCA and LAML, the mutation frequency of the OCGs was significantly higher than that of the target genes. For the OCGs, OV had the lowest average mutation frequency (0.40%), and UCEC had the highest (6.20%).

In summary, these results indicated that TSGs had the highest mutation frequency in most tumour types, and the OCGs were the second. The essential genes had the lowest mutation frequency in all tumor types.

### Network properties

To explore the network properties, we mapped the five gene sets onto human PPI networks and obtained the 48 TSG proteins, 49 OCG proteins, 161 target proteins, 141 essential proteins, and 12,315 other proteins. Then, we calculated four properties for each node in the network, including the degree, betweenness, clustering coefficient and shortest-path distance. To compare the network properties among the five sets of genes, we performed the K-S tests.

### TSGs and OCGs tended to have higher degree and betweenness

**Figure 2:**
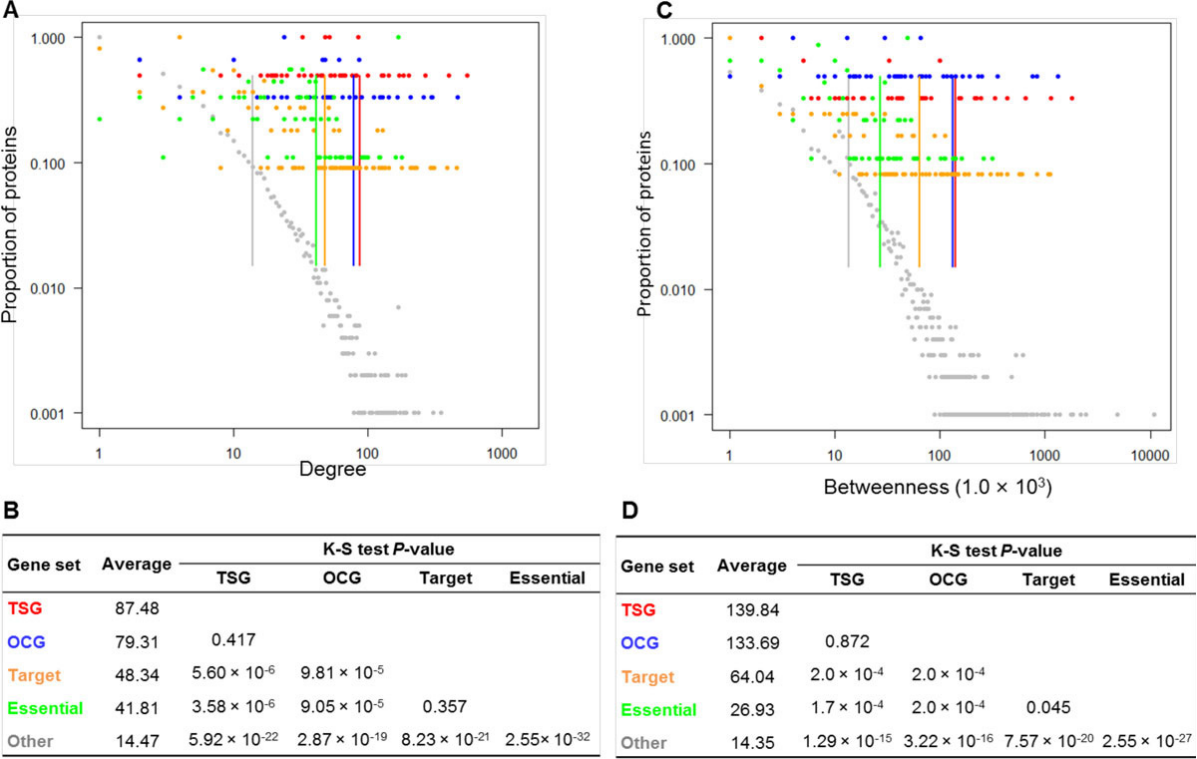
**Comparison of degree and betweenness of five protein sets**. A) Degree distribution. B) Summary of the average degree and the corresponding *P*-values of the Kolmogorov-Smirnov (K-S) tests for any two protein sets. C) Betweenness distribution. D) Summary of the average betweenness (1.0 × 10^3^) and the corresponding *P*-values of the K-S tests for any two protein sets.

Figure 2A shows the degree distributions for the five protein sets while Figure 2B contains their average degrees and K-S test *P*-values. The average degree of the TSG proteins was 87.48, which was significantly higher than that of the target proteins (48.34, *p *= 5.60 × 10^-5^), essential proteins (41.81, *P *= 3.58 × 10^-6^), and other proteins (14.47, *p *= 5.92 × 10^-22^). Similarly, the average degree of the OCGs was 79.31, which was also significantly higher than that of the target proteins (*p *= 9.81 × 10^-5^), essential proteins (*p *= 9.05 × 10^-5^), and other proteins (*P *= 2.87 × 10^-19^). However, we did not observe any significant difference between TSG proteins and OCG protein (*p *= 0.417). The average degrees of the TSGs and OCGs were approximately 2.0 times that of the target proteins and essential proteins and about 6.0 times that of the other proteins. The latter ratio is higher than that (3.1 times) found in cancer proteins in a previous study [27].

Figure 2C shows the betweenness distributions and Figure 2D contains the average value and K-S test *P*-values for the five protein sets. The results for the betweenness were consistent with those for the degree. These observations indicated that TSG proteins and OCG proteins had the highest degree and betweenness in the human PPI network compared to other proteins.

### TSGs and OCGs tended to have a lower clustering coefficient

**Figure 3:**
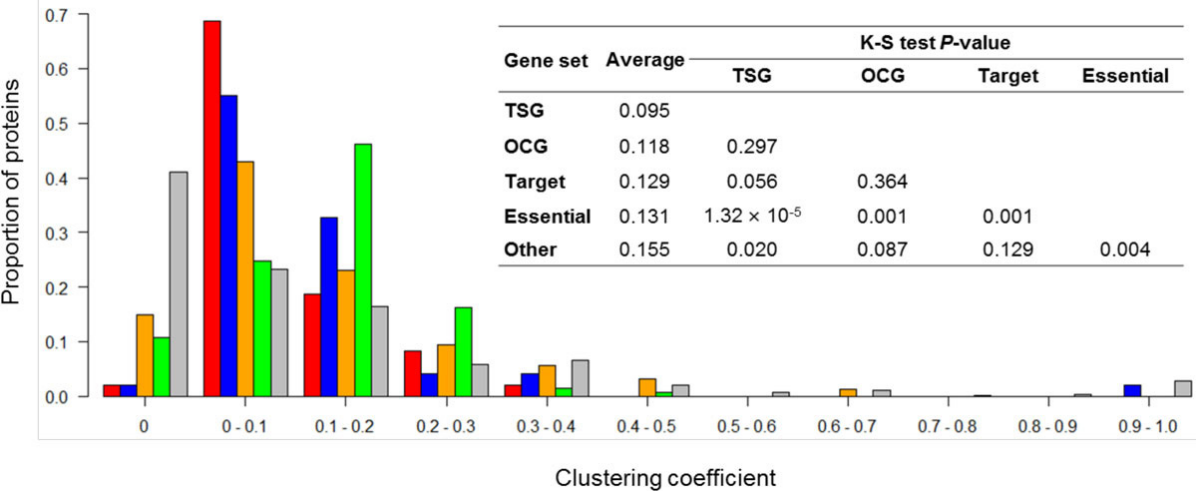
**Distribution of clustering coefficient of five protein sets**. The inserted table summarizes the average value of clustering coefficient for each protein set and the corresponding *P*-values based on the Kolmogorov-Smirnov (K-S) tests for any two protein sets.

For each node, the clustering coefficient reflects the connectivity among its interactors. The higher the clustering coefficient, the higher the connectivity of its neighbors has. Figure 3 shows the distribution of the clustering coefficient values, the average value of each protein set, and the K-S test *p*-values among the five protein sets. The average clustering coefficient of the TSG proteins was 0.095, which was significantly lower than that of the essential proteins (0.131, *p *= 1.32 × 10^-5^) and the other proteins (0.155, *p *= 0.020). Similarly, we found that the average clustering coefficient of the OCG proteins was 0.118, which was significantly lower than that of the essential proteins (*p *= 0.001), though only slightly lower than that of the other proteins (*p *= 0.087). We also found that the clustering coefficient of the essential proteins was significantly lower that of the other proteins (*p *= 0.004). To obtain the detailed distribution of clustering coefficients, we separated the clustering coefficients into different bins with an interval of 0.1 and calculated the proportion of the proteins in each bin. We found that, the proportion of the TSG proteins (68.8%) was higher than that of the OCG proteins (55.1%) at bin (0-0.1]. In contrast, at bin (0-0.2], the proportion of the TSG proteins (18.8%) was lower than that of the OCG proteins (32.7%).

### TSGs and OCGs tended to have shorter shortest-path distance

**Figure 4:**
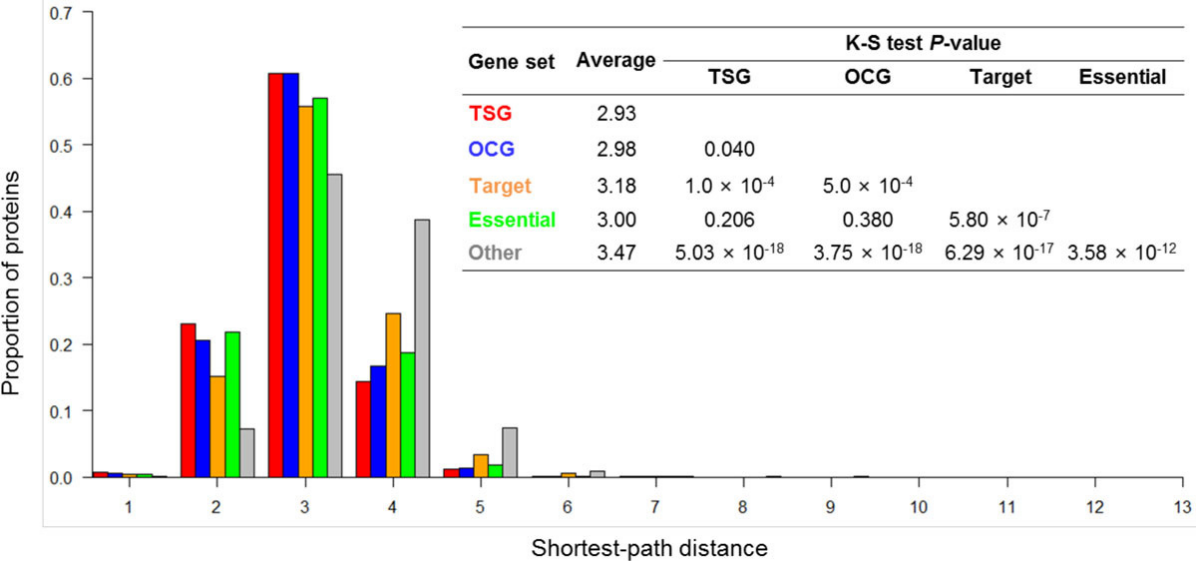
**Distribution of shortest-path distance from five protein sets to the other nodes in human protein-protein interaction network**. The inserted table summarizes the average value of shortest-path distance from each protein set to the rest nodes in human protein-protein interaction network and the corresponding *P*-values based on the Kolmogorov-Smirnov (K-S) tests for any two protein sets.

For each node, the shortest-path distance (SPD) was calculated from the node to all other nodes in the human PPI network. To summarize the measure, we utilized the average value of all shortest path distances to represent its shortest-path distance to others. Figure 4 shows the distribution of the SPD values, the average value of each protein set, and K-S test *p*-values among the five protein sets. The average shortest-path distance of the TSG proteins was 2.93, which was significantly shorter than that of the target proteins (3.18, *p *= 1.0 × 10^-4^), or the other proteins (3.47, *p *= 5.03 × 10^-18^). Interestingly, the average shortest-path distance of TSG proteins (2.93) was slightly lower than that of OCG proteins (2.98, *p *= 0.040). The average shortest-path distance of target proteins (3.18) was significantly longer than that of the essential proteins (3.00, *p *= 5.80 × 10^-7^) but significantly shorter than that of the other proteins (3.47, *p *= 6.29 × 10^-17^). While the proportion of shortest-path distances at each distance varied between the different sets, there were still a few similarities. In detail, from the shortest-path distance distribution at each distance, the proportion of proteins of different sets had much difference. For example, most proteins in each protein set have a shortest-path distance of 3.

### From targets to TSGs or OCGs in the human PPI network

**Figure 5:**
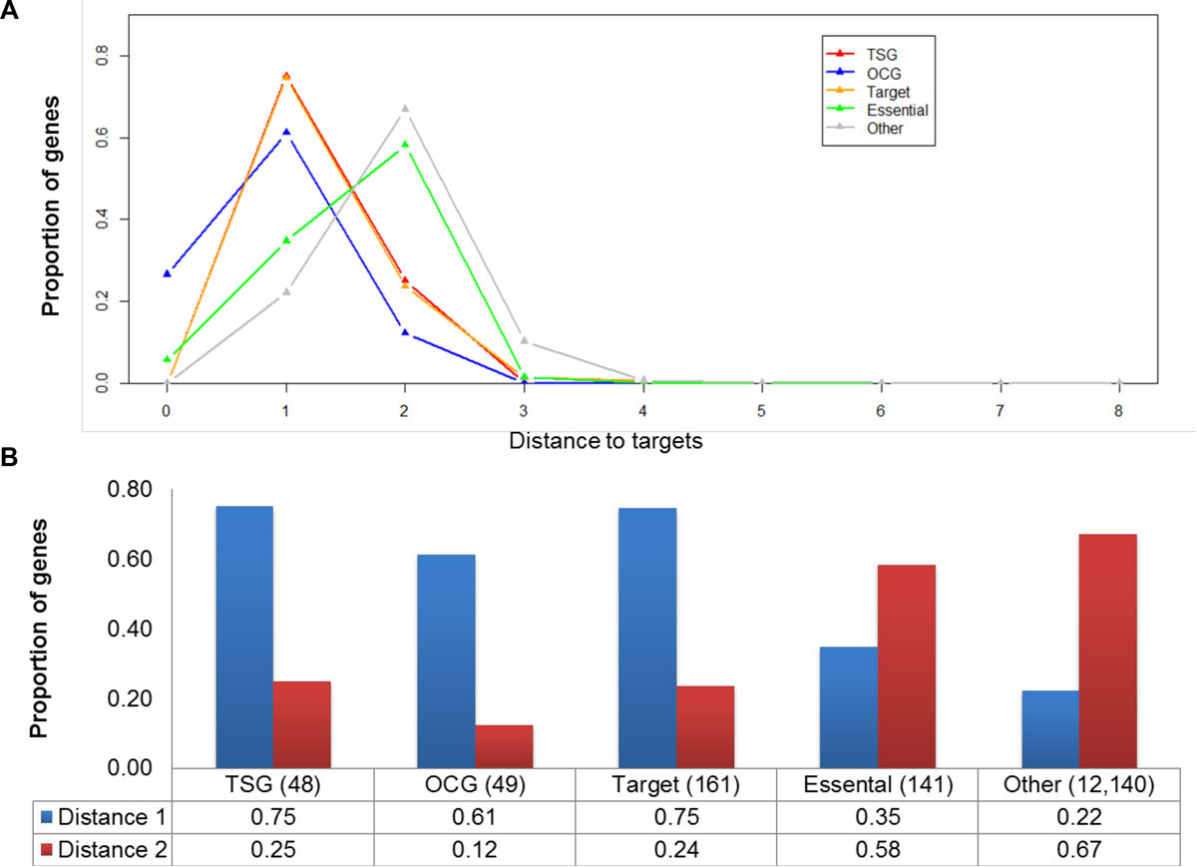
**Network-based relationship between target proteins to other four protein sets**. A) Distribution of shortest-path distance of five gene sets. B) Protein proportion at the shortest-path distance 1 and 2 from target proteins to TSG proteins or OCG proteins.

Most drugs exert their therapeutic actions through interactions with specific protein targets. Moreover, the TSGs and OCGs play important roles in the cancer development. Then, we compared the shortest-path distances from targets to TSG proteins or OCG proteins with the shortest-path distances from targets to essential proteins and other proteins. Figure 5A shows the fraction of each protein set in the drug target neighborhood with a measure of shortest-path distance from zero to eight. Among the 161 drug target proteins, 13 also belong to the OCGs and 8 belong to essential proteins. The rest of the OCG proteins (73%) and all TSG proteins (100%) were enriched at the shortest-path distances 1 and 2 from target proteins, which is consistent with the previous results of drug targets to cancer genes [31]. Additionally, most of the TSG proteins (75%), OCG proteins (61%), and target proteins (75%) had direct interactions with protein targets while other proteins (22%) had less direct interactions with protein targets (Figure 5B).

In summary, compared to the target proteins, essential proteins, and other proteins, both TSG and OCG proteins tended to have higher degrees, higher betweenness, lower clustering coefficients, and shorter shortest-path distances. Moreover, the TSG and OCG proteins did not have a significant difference with perspective of network topological properties. Both TSG proteins and OCG proteins tended to have more direct interactions with target proteins.

### TSGs and OCGs are highly connected

To further understand the relationship between TSG and OCG proteins in the local network organization and environment, we hypothesized that exploring TSG and OCG network would provide some novel insights. Then we generated one TSG-OCG network starting from the human PPI networks, 50 TSG proteins, and 50 OCG proteins.

**Figure 6:**
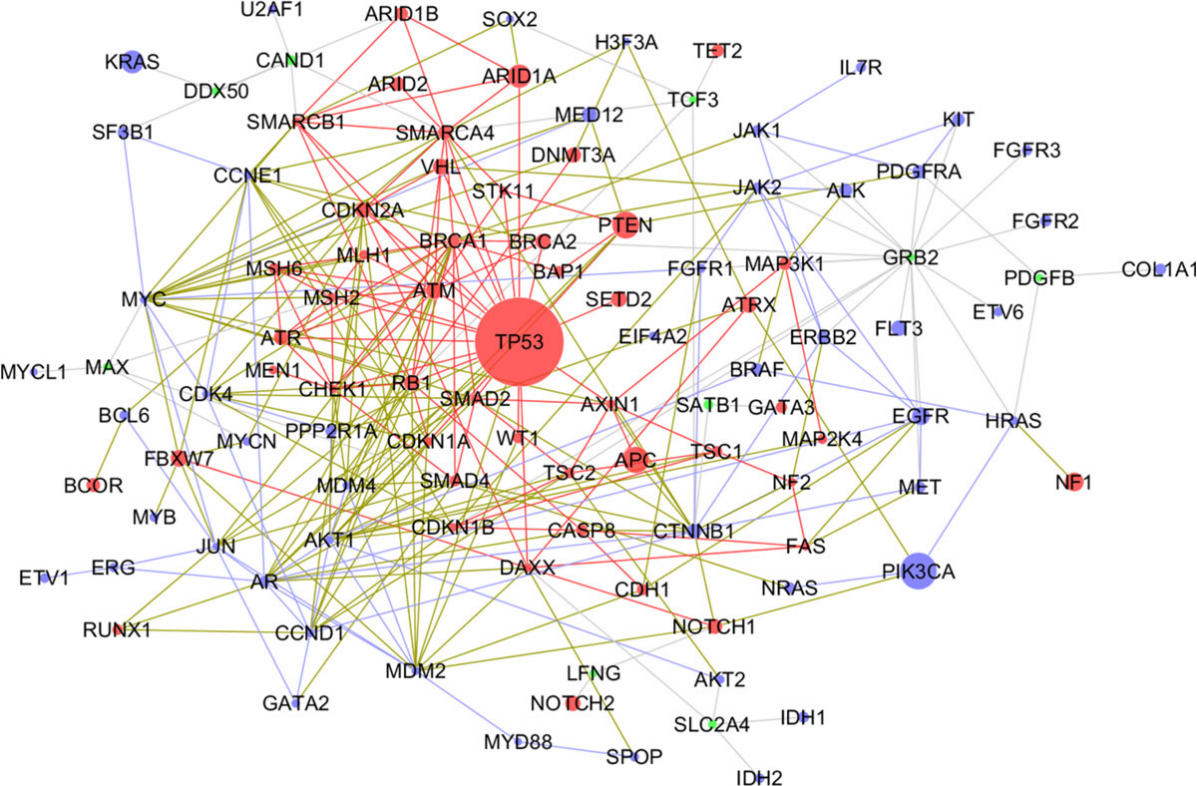
**TSG-OCG network**. Node color indicates the different protein sets: red for TSG proteins, blue for OCG proteins, and green for linkers that could link TSG proteins and OCG proteins. Edge color indicates protein-protein interaction among different protein sets: red for the interactions among TSG proteins, blue for the interactions among OCG proteins, dark green for the interactions between TSG proteins and OCG proteins, and gray for the interactions between linkers and OCG proteins or TSG proteins. Node size is corresponds to the mutation frequency in Pan-Cancer samples. The larger the node, the higher the frequency was.

The TSG-OCG network consisted of the 106 nodes and 303 edges (Figure 6). Among the 106 nodes, 48 belonged to the TSG proteins, which accounted for 96% of all the TSG proteins; 49 belonged to the OCG proteins, which accounted for 98% of all the OCG proteins; and 9 were linkers. The composition of the network indicated that the TSG-OCG network mainly consisted of the TSG and OCG proteins. Among the 303 edges, 89 links occurred among 42 TSG proteins, 51 among 36 OCGs, 117 among the 71 proteins (38 TSGs and 33 OCGs), and 46 between 9 linkers and 15 TSGs or 26 OCGs. Thus, 257 edges (84.8%) existed among TSGs and OCGs, suggesting that the TSG proteins and the OCG proteins were highly connected to each other in the context of protein-protein interaction networks. Moreover, the proportion of these links between the 38 TSGs and 33 OCGs (38.7%) were higher than that of interactions among the TSGs (29.5%) and that of interactions among OCGs (16.9%), respectively. Most of the TSGs (38, 79%) had at least one edge with OCGs. Similarly, most of the OCGs (67%) had at least one edge with TSGs.

To further explore the joint contribution of mutations in TSGs and OCGs, we integrated the mutation frequency of Pan-Cancer samples in each gene with the TSG-OCG network (Figure 6). The bigger node size represents the higher percentage of samples with mutations in Pan-Cancer project. The mutation frequency of the 106 genes encoding the 106 nodes in the TSG-OCG network ranged from 0.33% to 46.15% with the average value of 3.14%. We further examined the correlation between the mutation frequency and degree of proteins using Pearson's correlation. We found that the mutation frequency and degree of proteins had a significant correlation (r = 0.30, *P*-value = 0.002). The observation indicated that the higher direct associations among these genes with higher mutation frequencies might contribute to the cancer development jointly. For example, TP53 had the highest mutation frequency in all samples and had 26 interactors. Among them, 21 were TSGs and four OCGs. Among the 21 TSGs, gene *PTEN *is another TSG gene with higher mutation frequency (11.27%), which might indicate that they might contribute to the cancer development together. In fact, several studies have demonstrated that that the *PTEN *and *T53 *genes jointly participate in the carcinogenesis o may malignancies [32]. Similarly, another example is the gene *ARID1A *that has an association with TP53 and had a higher mutation frequency (11.27%). One previous study has shown that one mutation in the gene associated with mismatch repair efficiency and normal p53 expression [33].

## Discussion

Cancer is a genetically complex disease, which involves the combined functions of tumor suppressor genes (TSGs) and oncogenes (OCGs). TSGs and OCGs jointly play important roles in the cancer development through loss of function or gain of function. Most of them cannot trigger the cancer development by themselves. Numerous studies about genetic alterations of TSGs and OCGs, especially OCGs, have led to the identification of drug targets for cancer treatment. However, the identification of novel drug targets has become more challenging even though genome-wide sequencing data provide thousands of mutations. Therefore, development of novel approaches for identification of novel drug target is mandatory. To facilitate the development of novel approaches, in this study, we comprehensively compared TSGs and OCGs from the perspectives of somatic mutation and network properties. These broad comparative results allow us to address several questions that might be useful for the development of new methods: 1) Do TSGs and OCGs have similar or different mutation frequency patterns? 2) How do they relate to each other? 3) How do they relate to cancer drug target? 4) Do the TSGs and OCGs tend to link closely to each other? The results indicated that while the TSGs and OCGs had different mutation patterns, they had similar network characteristics. They were also not only related to each other closely, but also to cancer drug targets.

In this study, we mainly focused on the examination of the mutation patterns of TSGs and OCGs from the whole-genome wide data in the Pan-Cancer project [23]. It was different from the purpose of the Pan-Cancer analysis project. The study of Pan-Cancer analysis presents the data and analytical results for point mutations and small insertions/deletions from 3,281 tumours across 12 tumour types as part of the TCGA Pan-Cancer effort. They illustrated the distributions of mutation frequencies, types and contexts across tumour types, and establish their links to tissues of origin, environmental/carcinogen influences, and DNA repair defects. However, they did not go further to examine the mutation patterns of TSGs and OCGs. In this study, we separated the mutation data of TSGs and OCGs from the rest genes and performed a comparison of five gene sets. We found that the TSGs had the highest mutation frequency in most tumour types and the OCGs second. The results might be interpreted by the theory that the gain-of-function mutations that convert proto-oncogenes to oncogenes acts dominantly while the loss-of-function mutation in tumor suppressor genes acts recessively. In addition, we observed that the essential genes had the lowest mutation frequency in all tumor types, which might reflect the fundamental roles in the survival of the essential genes.

However, we did not dive further to study the consequence of or causal relation to mutations for the function roles of TSGs and OCGs. As we known that TSGs and OCGs have different roles during the cancer development. However, it is not very clear how they work together. It will be very interesting and useful to further study the association between the mutation frequency and the roles of TSGs and OCGs. For example, we observed that the mutation frequency of TSGs was about two times of that of OCGs. It is not clear whether or not this mutation frequency difference influence or linked to their functional roles in the pathogenesis of cancer. Moreover, it is very challenging to assess the association between the mutation frequency difference and functional roles of TSGs and OCGs by both computational and experimental examination.

In this study, we compared the drug target genes with TSGs and OCGs in the view of mutation frequencies and network properties. We found drug target genes generally tend to have less mutations compared to TSGs and OCGs and also have lower degrees. These results suggested that the genetic contribution of drug target genes is not strong as TSGs and OCGs. Besides, we found both TSG and OCG proteins tended to have direct interactions with cancer drug target proteins. However, we did not further examine if the drug targets either suppress actions on oncogene activity or restore TSG functions through direct interaction or indirectly interactions. It might be very interesting to further examine if the mutations in OCGs or TSGs are necessary for both the establishment and maintenance of protein-protein interactions, which might lead to the identification of logical drug targets. However, to map the mutation to proteins for detecting the mutation-specific perturbations at the network level need much efforts including the development of protein structure-guided pipeline for extracting interacting protein sets specific to a particular mutation, which is beyond of the scape of this study. In the future, we will integrate the protein structure information with mutation information in the context of PPI network to further understand the connection of TSG and OCG proteins in the cancer development.

The study was mainly based on the data coming from both public data and predicted results. As most of the computational biology studies, it is very challenging to obtain the error-free or complete data. Therefore, in the analysis process, there still have several steps we could improve in the future, including the selection of gene sets, specification of protein function association data, and mutation data of cancer with less bias. For gene sets, we chose the genes with high confidence for analysis. The data set used here are far from complete and error-free. For the protein associations, we utilized the PPI data from PINA database, which includes the physical association, genetic association, and enzymatic reaction curated from six other databases. It is not clear about how these mutations alter the interaction relationship with their partners. For mutation data from cancer patients, we mainly utilized the data from TCGA, which might be biased by sequencing depth, platform, and sample size. However, our analysis still provided statistically significant characteristics of somatic mutations and networks of TSGs and OCGs. The list of TSGs and OCGs is updated frequently based on different methods. Therefore, the characteristics of TSGs and OCGs under investigation will not be exactly the same as those we concluded here. However, the tendencies we obtained in this study might provide some clues for further investigation of functional roles of TSGs and OCGs in carcinogenesis and identification of novel drug targets.

## Conclusion

In this study, we explored the somatic mutation and network characteristics of TSGs and OCGs. Based on the mutation data from Pan-Cancer project, we found that the TSGs had the highest mutation frequency. Based on the human protein-protein interaction network, we found that TSG proteins and OCG proteins had similar global network topological characteristics and that the TSGs, OCGs, and drug targets had a tendency to interact with each other. Integration of mutation frequency with TSG-OCG network provided insights that TSGs and OCGs might jointly contribute to the cancer development. In summary, this study first comprehensively investigated TSGs and OCGs from the perspective of genetics and networks, which provides novel insight into the roles of TSGs and OCGs in cancer development and treatment.

## Competing interests

The authors declare that they have no competing interests.

## Authors' contributions

KZ, QL, JS conducted data collection and data analysis. YZ participated the data analysis. JS and HX conceived and designed the study. KZ, JS, YZ, CT, ZZ, and HX contributed to the writing of the manuscript. All authors read and approved the final manuscript.

## Declaration

The publication funding came from Cancer Prevention & Research Institute of Texas (CPRIT R1307).

This article has been published as part of *BMC Genomics *Volume 16 Supplement 7, 2015: Selected articles from The International Conference on Intelligent Biology and Medicine (ICIBM) 2014: Genomics. The full contents of the supplement are available online at http://www.biomedcentral.com/bmcgenomics/supplements/16/S7.
